# Motivation to change and perceptions of the admission process with respect to outcome in adolescent anorexia nervosa

**DOI:** 10.1186/s12888-015-0516-8

**Published:** 2015-07-02

**Authors:** Simona Hillen, Astrid Dempfle, Jochen Seitz, Beate Herpertz-Dahlmann, Katharina Bühren

**Affiliations:** Department of Child and Adolescent Psychiatry, Psychosomatics and Psychotherapy, University Hospital of the RWTH Aachen, Neuenhofer Weg 21, 52074 Aachen, Germany; Institute of Medical Biometry and Epidemiology, Philipps-University Marburg, Marburg, Germany; Institute of Medical Informatics and Statistics, Christian-Albrechts-University Kiel, Kiel, Germany

**Keywords:** Motivation to change, Readiness to change, Anorexia nervosa, Adolescence, Weight gain, Outcome, Treatment, Perception of admission process, Stages of change

## Abstract

**Background:**

In patients with anorexia nervosa (AN), there is evidence that readiness to change is an important predictor of outcome with respect to weight gain and improvement in eating disorder psychopathology. In particular, young patients are characterized by a low level of motivation for recovery and perceive more coercion at hospitalization. Thus, a better understanding of the variables that influence readiness to change and perception of the admission process in adolescent AN may help to support patients in initiating change and staying motivated for treatment.

**Methods:**

In 40 adolescent patients diagnosed with AN according to DSM-IV criteria, we assessed in a prospective clinical cohort study the motivation to change using the Anorexia Nervosa Stages of Change Questionnaire (ANSOCQ) at admission to inpatient treatment, in week 9 after admission and at discharge. Additional variables were assessed, including depressive symptoms (Beck Depression Inventory, BDI), eating disorder-specific psychopathology (Eating Disorder Inventory, EDI-2), body mass index (BMI) and the percentage of expected body weight (%EBW). The patients’ perceptions of the admission process and their perceived need for hospitalization were assessed using a self-report scale developed by Guarda et al. (2007).

**Results:**

Younger patients perceived more coercion than older patients did. Low %EBW and more severe eating disorder-specific psychopathology were associated with a greater perceived need for hospitalization. Moreover, low %EBW at admission and a longer duration of illness were accompanied by a greater motivation to change at admission, whereas more severe eating disorder psychopathology was associated with a low motivation to change. The motivation to change increased significantly between admission and discharge. Patients with a greater motivation to change at admission exhibited a higher weekly weight gain during treatment but did not show better outcome in eating disorder-specific psychopathology and depression.

**Conclusions:**

Motivation to change is an important predictor of short-term outcome with respect to weight gain trajectory during treatment of adolescent AN. As patients with a higher BMI at admission and those with more severe eating disorder-specific symptoms seem to be less motivated to change, the crucial issue of motivation to change should be addressed with these patients during the therapeutic process.

## Background

Anorexia nervosa (AN) is characterized by a high risk of chronicity [1] and a five-fold increased mortality rate [2]. The onset of an eating disorder during adolescence often has a substantial impact on physical and psychological development [3, 4] with the potential for severe medical and neuropsychiatric consequences in adulthood [5]. Approximately half of patients require at least one readmission [6], with nearly one quarter of patients relapsing within the first year after discharge [7, 8].

The ego-syntonic quality of the eating disorder and patients’ insufficient awareness of their conditions contribute to their reluctance to recover. The core symptoms of AN, such as a fear of gaining weight and body image disturbances, might also reinforce the low willingness of patients to introduce any change to their state of illness [9]. The pretreatment level of motivation to change was found to be a relevant predictor of treatment outcomes with respect to weight gain and improvements in eating disorder psychopathology [10–12]. A low readiness to change was associated with premature termination of treatment [13] and relapse [14, 15]. Several studies have highlighted the association between a patient’s motivation to change at the beginning of treatment and the clinical features at first presentation, such as body mass index, depression and body dissatisfaction (for an overview, see [16]).

In particular, young patients with eating disorders are often characterized by a low level of motivation for recovery [17]. However, only a few studies have investigated readiness to change in this age group. Geller et al. [18] reported a negative relationship between readiness to change and severity of eating pathology at different stages during inpatient and outpatient treatment in sixty-five 12- to 18-year-old girls with an average BMI of 19.4 kg/m^2^. Low motivation to change was associated with a longer duration of residential treatment in 65 females aged 14–19 years and slower weekly weight gain in adolescent and adult patients [11, 19]. In a study of 49 female adolescents (average age of 14.3 years), poor weight maintenance nine months after discharge from inpatient treatment was observed in patients with a low motivation to change. Another study found a higher risk for hospital readmission 6–9 months after hospital discharge in 70 females between 13 and 19 years of age [20].

The perceived need for hospitalization is low, and patients are often admitted to hospital under pressure from clinicians and caretakers. Adolescents perceive more coercion and less agreement than adult patients do [21]. Moreover, there is evidence that adult patients who are involuntarily admitted to treatment seem to subsequently accept the necessity of such treatment [22] and gain an equivalent amount of weight compared with individuals who are hospitalized voluntarily [23].

Because of a dearth of studies on adolescent AN, the aims of the present study were as follows: (1) to evaluate an association between patient characteristics, motivation to change at admission and perception of the admission process, (2) to evaluate whether the motivation to change varied over the course of treatment, and (3) to evaluate whether motivation to change at admission had an impact on treatment outcomes with respect to weekly weight gain and improvements in eating disorder-specific psychopathology.

We hypothesized that patients with more severe eating disorder psychopathology and more feelings of coercion at admission would have a lower motivation to change, and we assumed that motivation to change increased during treatment and was positively associated with weekly weight gain. Younger patients and those with a less severe course of illness were expected to have a lower perceived need for hospitalization.

## Methods

### Participants

All female adolescents between 10 and 18 years of age who were consecutively admitted to inpatient AN treatment at the Department for Child and Adolescent Psychiatry, Psychosomatics and Psychotherapy of the RWTH Aachen University Hospital between March 2011 and October 2012 were asked to participate in this study. All patients and their parents or legal guardians provided written informed consent. The study was approved by the local ethics committee (Ethics Committee of the RWTH Aachen Faculty of Medicine).

All participants met the DSM-IV diagnostic criteria [24] for AN at the time of admission. The weight threshold for inclusion in the study was a BMI below the 10th percentile (based on a large German reference set [25]) according to an international convention for the definition of adolescent and childhood AN [26, 27] and corresponding to the German guidelines for eating disorders [28]. For the vast majority of the patients, it was their first AN-related hospital admission (two patients had one previous hospitalization, and one patient had two prior hospital stays because of the eating disorder). All patients were admitted voluntarily and were primarily treated in an inpatient treatment setting for medical observation and stabilization, which was followed by a day patient setting for most patients. An identical multimodal multidisciplinary treatment program [8, 29] was used in both settings (inpatient and day patient treatment), including weight restoration (for all patients, a minimum weight gain of 300 g/week was intended), individual and group nutritional counseling, cognitive-behavioral individual and group therapy, individual family sessions, and a group psychoeducation program for parents (for further details, see [8]). The patients were discharged when they had maintained their target weight (approximately the 15th- 20th age-adjusted percentile) for 2 weeks.

### Assessments

The patients’ motivation to change was assessed using the Anorexia Nervosa Stages of Change Questionnaire (ANSOCQ [30, 31]), which was translated into German. The German version was validated by Pauli et al. (publication in preparation). The ANSOCQ is a self-report questionnaire with 20 items (scored on a scale of 1 to 5 for each item) that assesses various aspects of AN-specific symptomatology, including attitudes toward body shape and weight, eating behavior, weight control strategies and emotional and social difficulties. It is based on the stages of change model developed by Prochaska and DiClemente [32]. The total score can be assigned to one of five motivational stages, defined as precontemplation, contemplation, preparation, action and maintenance, with high total scores indicating a high level of motivation to change. This questionnaire has been applied in several previous studies of AN [12, 33]. The internal consistency, test-retest reliability and validity of the questionnaire have been demonstrated previously [30, 31]. In our sample, the internal consistency of the total ANSOCQ score was also good, with Cronbach’s alpha estimated at 0.93 at admission, 0.95 in week 9 and 0.96 at discharge [34, 35].

The patients’ perceptions of the admission process and of the need for hospitalization were assessed using a 13-item self-report scale developed by Guarda et al. [21] based on the structured Mac Arthur Admission Experience Interview [36, 37]. The first 12 items pertain to three subscales (4 items each with scores from 1 to 5): 1) perceived coercion regarding the decision to be admitted, 2) pressure from others to be hospitalized and 3) procedural justice during the admission process from the patient’s perspective. Thus, scores for each subscale can range from 4 to 20, with higher scores indicating more perceived coercion, more pressure and greater procedural justice. The last item assesses the patient’s perceived need for hospitalization using a rating scale ranging from 1 (= no perceived need) to 5 (= perceived need). Good internal consistency with Cronbach’s alpha coefficients for perceived coercion, pressure, and procedural justice estimated at 0.91, 0.65, and 0.82, respectively, has been reported by the authors [21]. The Cronbach’s alpha coefficients in our sample were 0.90 for perceived coercion, 0.72 for pressure and 0.53 for procedural justice [34, 35].

The expert form of the Structured Interview for Anorexic and Bulimic Disorders (SIAB-EX [38]) was used at admission to establish a diagnosis of AN according to DSM-IV. The SIAB-EX is a semi-standardized interview to determine the prevalence and severity of specific eating disorder-related symptoms. The SIAB-EX also differentiates between the two subtypes of AN in DSM-IV (restrictive and binge-purging subtypes). Eating disorder-specific psychopathology was evaluated using the German version of the Eating Disorder Inventory (EDI-2, German version [39]), a self-report questionnaire with 91 items. Higher scores indicate more severe psychopathology; both the total score and the two subscales most relevant to the core symptoms of AN (drive for thinness and body dissatisfaction) were considered. Depressive symptomatology was assessed using the Beck Depression Inventory (BDI-2, German version [40]), which is a self-report questionnaire with 21 items.

Assessments were always performed during the first week following admission, during week 9 of treatment and at discharge. At admission and discharge, BMI, EDI-2, BDI-2 and ANSOCQ were assessed. During week 9 of treatment, only the ANSOCQ was used. Clinical characteristics such as age at admission, illness duration, premorbid weight and length of hospital stay were recorded. The percentage of weight loss was calculated using premorbid weight and weight at the time of admission. The BMI percentiles and the percentage of expected body weight (%EBW) were calculated on the basis of a large German population-based normative data set [25]. The rate of weekly weight gain was calculated as the difference between weight at discharge and admission divided by treatment duration in weeks. The rate of change in %EBW was defined analogously.

### Statistical analysis

To compare the participants with patients who refused to participate with respect to variables such as age, BMI at admission, illness duration and percentage of weight loss (only these variables were available for non-participants), we used Student’s unpaired two-sample t-tests together with confidence intervals of the mean difference. For our first aim, the identification of variables associated with motivation to change at admission and perceptions of the admission process, model selection using a stepwise multiple regression was performed. The ANSCOQ total score or the total scores of the subscales “perceived coercion”, “perceived pressure”, “perceived procedural justice” and “perceived need for hospitalization” were used as dependent variables. We investigated age, %EBW, illness duration, percentage of weight loss and EDI-2 (total or subscales) and BDI-2 total scores at admission as independent variables. The model with the largest adjusted R^2^ was considered the best model. Second, we compared the means of quantitative variables (EDI-2, BDI-2, and ANSOCQ) between admission and discharge using paired Student’s t-tests and investigated the change in ANSOCQ over time using a linear mixed model (Bates, e-print) to incorporate the week 9 assessment and to consider the different durations of treatment until discharge (random intercept model). We also investigated potential predictors of improved motivation to change by using ANSOCQ at discharge as an outcome variable in a multiple regression model. For our third aim, we performed multiple regression analyses in the same manner as described above to investigate the association between motivation to change at admission and perception of the admission process with respect to physical (rate of change in %EBW and weekly weight gain (in g)) and cognitive (EDI-2 and BDI-2 at discharge) outcome variables. Because there were some missing values for EDI-2 and BDI-2 at discharge, we used both a complete case analysis and multiple imputation [41]. All statistical analyses were performed using R [42].

## Results

### General characteristics

Of the 47 consecutively admitted patients, seven patients did not agree to participate in the study. The patients who agreed to participate in the study and the patients who refused to participate were similar with respect to age (95 % confidence interval (CI) for a difference in mean age of −2.1 to 2.3 years), %EBW at admission (95 % CI for a difference in mean %EBW of −3.4 to 4.9) and duration of illness (95 % CI for a difference in months of −10.1 to 8.8) (all *p* > 0.1). Six patients participated in assessments only at admission but not at discharge; three patients did not participate for organizational reasons (e.g., the patients decided to be discharged without prior notice), two patients were discharged against medical advice, and one patient refused to participate in the discharge assessment. These six patients did not differ from the 34 others with respect to any of the baseline variables (all *p* > 0.2). The general characteristics of the 34 patients are presented in Table 1. Thirty-seven patients (92.5 %) were diagnosed with the restricting subtype of AN, and three patients (7.5 %) were diagnosed with the binge-purging subtype. The EDI-2 (total and relevant subscales), BDI-2 and ANSOCQ scores at admission and during the course of treatment are presented in Table 2.

**Table 1:** General characteristics at admission, discharge and during treatment

	Mean (SD) or n	Range
Admission		
percentage of weight loss (%)^a^	22.7 (836)	6.1-41.4
duration of illness (months)	10.1 (7.8)	3.0-40.0
age(years) at admission	15.1 (1.6)	10.9-18.7
BMI (Kg/m^2^) at admision	15.7 (1.2)	13.3-18.3
% EBW at admision	77.6 (5.4)	66.7-87.5
Treatment		
duration of tratment (weeks)	15.1 (5.5)	5.4-30.1
weight gain (g/week)	459 (229)	184-1188
BMI gain (g/week)	0.2 (0.1)	0.1-0.4
%EBW gain per week	0.8 (0.4)	0.3-2.1
Discharge		
BMI (kg/m^2^) at discharge	18.0 (1.0)	14.3-19.6
%EBW at disharge	88.5 (4.4)	74.7-95.8

^a^percentage of weight loss refers to weight at the onset of the disorder

BMI = body mass index, %EBW = percent expected body weight

**Table 2:** Psychopathological characteristics and motivation to change at the different time points and patient´s perception of the admission process and perceived need for hospitalization

	Admission mean (SD)	Week 9 mean (SD)	Discharge mean (SD)	p value^a^
EDI-2	270.9 (66.6)		255.5 (78.1)	0.36
-subscale drive for thinness	27.3 (9.8)		24.6 (10.2)	0.35
-subscale body dissatisfaction	35.9 (9.7)		36.0 (13.7)	0.75
BDI-2	21.9 (11.0)		12.1 (10.0)	0.002
ANSOCQ^b^	50.2 (14.8)	55.9 (20.2)	71.6 (23.0)	<0.0001
perceived coercion	15.2 (4.2)			
perceived pressure	8.9 (4.0)			
perceived preocedural justice	14.7 (2.9)			
perceived need for hospitalization	3.6 (1.0)			

^a^paired t-test admission to discharge

^b^n at admission = 40, n at week 9 = 29 (9 patients were discharged before or in week 9; these are not included here and only the ANSOCQ at discharge is given), n at discharge = 35

EDI-2 = Eating disorder inventory 2; BDI-2 = Beck depression inventory 2; ANSOCQ = Anorexia nervosa stages of change questionnaire; perception of the admission process according to Guarda et al. (2007)

### Patient perceptions of the admission process and the perceived need for hospitalization

Perceptions of the admission process evaluated according to the questionnaire of Guarda et al. [21] are indicated in Table 2. Younger patients (*p* = 0.005) experienced higher levels of perceived coercion. Younger age and lower weight loss at admission together accounted for 23 % of the variance in the perceived coercion at admission. Patients with a higher %EBW (*p* = 0.01) reported higher levels of perceived pressure. %EBW alone accounted for 16 % of the variance in the perceived pressure at admission. Younger patients perceived significantly lower procedural justice at admission (*p* = 0.03), with age alone accounting for 12 % of the variance in the perceived procedural justice. Patients with a low %EBW (*p* = 0.004), a high percentage of weight loss (*p* = 0.003) and more severe eating disorder psychopathology (EDI-2 scores, *p* = 0.004) at admission reported a strong perceived need for hospitalization. Together, these variables accounted for 47 % of the variance in the perceived need for hospitalization.

### Motivation to change at admission

**Fig. 1:**
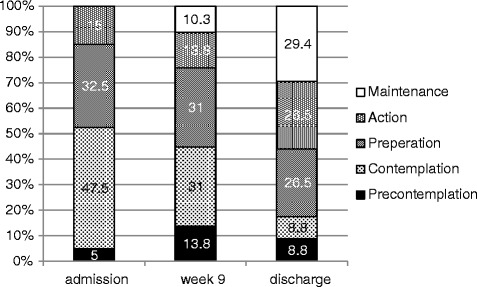
Overview of the distribution of the motivational stages at the different time points according to the ANSOCQ (Anorexia Nervosa Stages of Change Questionnaire)

At admission, the patients’ mean total ANSOCQ score was 50.2 (SD 14.8). Most patients were in the “contemplation stage” (39.6 %), followed by more than one quarter (27.1 %) who were in the “preparation stage” (Fig. 1). A low %EBW at admission (*p* = 0.002) and a longer duration of illness (*p* = 0.04) were accompanied by a greater motivation to change at admission, whereas more severe eating disorder psychopathology (EDI-2 scores; *p* = 0.0001) was linked to a lower motivation to change at admission. In our sample, the physical index of illness severity (measured by weight status (%EBW)) and the cognitive index of illness severity (measured by EDI-2 scores) at admission were negatively correlated (rho = 0.29), which may explain the inverse relationship of these indices to patients’ motivation to change. Together, %EBW at admission, the duration of illness and the severity of eating disorder-specific psychopathology explained 59 % of the variance in the motivation to change at admission. Considering the EDI-2 subscales drive for thinness (DT) and body dissatisfaction (BD) instead of the total EDI-2 score yielded similar results (p < 0.0001 for DT and *p* = 0.0002 for BD in models with %EBW at admission and duration of illness). Depressive symptoms were not significantly associated with ANSOCQ scores at admission (*p* = 0.5).

### Motivation to change during the course of treatment

The patients’ motivation to change increased significantly between admission and discharge (p < 0.0001, see Table 2). On average, patients’ motivation increased by 21.7 points (95%CI: 15.3 to 28.2), which corresponded to a shift to the next stage of change. During treatment, the ANSOCQ score increased by an average of 1.1 points per week (95%CI: 0.7 to 1.5, p < 0.0001, main effect of time in a linear mixed model; a model with time^2^ as a predictor did not provide a better fit). An overview of the distribution of the motivational stages at different times is presented in Fig. 1. Except for the score for motivation to change at admission, none of the variables at admission was related to motivation to change at discharge.

### Association with short-term outcomes

Patients with a greater motivation to change at admission exhibited more rapid weekly weight gain during treatment, as measured by both increases in %EBW per week (*p* = 0.008 for ANSOCQ in a model adjusted for age, %EBW at admission and duration of illness) and weekly weight gain (in kg) (*p* = 0.01, same adjusted model). Motivation to change (ß = 0.01), age (ß = 0.007), %EBW at admission (ß = −0.02) and illness duration (ß = −0.01) together accounted for 36 % of the variance in the %EBW increase. The same parameters (motivation to change (ß = 0.007), age (ß = 0.01), %EBW at admission (ß = −0.009) and illness duration before admission (ß = −0.008)) together accounted for 36 % of the variance in weight gain per week. An ANSOCQ score that was 20 points higher at admission (corresponding to one motivational stage) was associated with an additional weekly weight gain of approximately 132 g (95 % confidence interval: 33 g to 231 g). Outcomes in specific eating disorder psychopathology and depression (EDI-2 total or subscales and BDI-2 at discharge) were not associated with motivation to change at admission (all *p* > 0.7) in either a complete case analysis or a multiple imputation approach. The perceptions of coercion, pressure and procedural justice as well as the perceived need for hospitalization at admission were not significant predictors of weekly weight gain.

## Discussion

In our prospective clinical cohort study of motivation to change, we demonstrated for the first time that a greater motivation to change at the beginning of treatment resulted in a faster weekly weight gain in adolescent patients with AN during hospital treatment. Patients with a lower %EBW at admission and a longer duration of illness exhibited greater motivation to change; the reverse was true for individuals with more severe eating disorder-specific psychopathology. Younger patients perceived more coercion than older patients did. A higher %EBW and less severe eating disorder psychopathology were associated with a lower perceived need for hospitalization.

Similar to another study [43], the patients’ perception of the admission process and the perceived need for hospitalization were not correlated with their motivation to change and were not significant predictors for any of the outcome measures. Moreover, some authors have reported that many patients revise their perceptions of the need for hospitalization within the first weeks of treatment [21, 36]. In our study, patients with lower weight (%EBW) at admission, greater weight loss and more severe eating disorder symptoms perceived a greater need for hospitalization. We confirmed the results of a study by Guarda et al. [21], who reported significantly less procedural justice, higher levels of coercion and pressure, and a significantly lower perceived need for hospitalization in younger patients compared with adult patients.

In the present study, different patient characteristics were related to the patients’ motivation to change at the beginning of treatment. It is notable that physical symptoms of the eating disorder (measured by %EBW) and cognitive symptoms representing psychopathology (measured by the EDI - 2) were inversely correlated at admission. This negative relationship between the physical and cognitive index of illness severity has also been described in a recent study [44] that reported a greater drive for thinness and more body dissatisfaction in patients with a higher BMI. The authors suggest that the more pronounced eating disorder-specific psychopathology in these patients might result from wanting to become more underweight or comparing themselves with thinner eating disorder patients. Moreover, it must be considered that weight status (%EBW, weight loss) and illness duration are objective measures, while the questionnaires scores (EDI -2) are based on the patients’ subjective evaluations.

A longer duration of illness, greater weight loss and lower weight (lower %EBW) at admission were associated with a readiness to change. These results suggest that adolescent patients with more severe physical symptoms exhibit a stronger motivation to change. Similarly, Casanovas et al. [17] reported that a longer duration of illness was associated with a greater motivation to change in adolescents, and Geller et al. [45] identified initial weight status as an important variable for adult patients’ ambivalence toward treatment. Patients with longer illness duration and lower weight at admission may have experienced more starvation-associated symptoms that led to greater psychological strain and therefore had better insight into their illness and greater motivation to change compared with patients with better health states.

In contrast to these findings, more pronounced cognitive symptoms of the eating disorder were associated with a lower readiness to change (after controlling for BMI) in our study. Other very recent studies with adolescent AN patients [18, 46, 47] also reported that eating disorder-specific psychopathology, particularly drive for thinness and body dissatisfaction (measured by EDI – 2), was associated with a lower motivation to change. The latter finding might be explained by the high ego-syntonic quality of the disease, which leads to ambivalence toward treatment [48].

We found a significant increase in motivation corresponding to approximately one defined stage in motivation to change between admission and discharge, which is consistent with previous research [20, 33, 49]. This finding might result from different factors, such as psychological treatment, positive relationships with fellow patients and positive experiences in the patient’s social environment [50]. However, it is possible that the rather high ANSOCQ at discharge may also be influenced by the knowledge of imminent discharge that affected the participants’ statements. We did not identify any predictors of improvement in the motivation to change among the clinical characteristics available at admission. However, at discharge, only half of the patients were in the “action” or “maintenance” stage of change; the other half remained in the “contemplation” or “preparation” stages. This finding demonstrates the persistent ambivalence in whether to fully give up eating disorder behaviors after weight recovery in a significant subgroup of patients. Given the previously described importance of readiness for change [51], it may be beneficial to integrate interventions designed to enhance motivation in the treatment of patients with eating disorders. According to our results, younger patients and patients with more severe eating disorder symptoms or smaller weight loss had the lowest readiness to change. Unfortunately, a very recent review [51] and a meta-analysis [52] could not demonstrate an effect of motivational enhancement therapy or motivational interviewing in eating disorders; thus, more effective therapeutic interventions must be developed.

During the course of our study, a greater motivation to change at admission was followed by higher weekly weight gain. This result is consistent with the results of other studies of adolescent patients [11, 33, 53] that identified motivation to change as a predictor of weight gain during hospital treatment. In accordance with our results, McHugh et al. [33] reported a shorter treatment duration in patients with a greater motivation to change. However, the influence of motivation to change might be more important in adolescent patients at the beginning of treatment than in adults, who often suffer from AN symptoms for many years and undergo treatment several times. While Castro-Fornieles et al. [49] reported the stages of change at admission to be predictive of BMI at discharge in adolescents, Mander et al. [15] could not find any effect of motivation to change on BMI in an adult sample with a predominantly chronic course of AN (with a mean duration of 8.6 years of full syndromal AN).

Some methodological limitations must be considered. Our results pertain only to hospitalized patients. It is possible that these patients are more severely ill and that they therefore exhibit a stronger motivation to change and a greater need for hospitalization than non-hospitalized patients do. In addition, our results may apply only to adolescent female patients with a rather short duration of illness, who may have a stronger “readiness to change”. Our observational study design cannot explain any causal relationships. Moreover, we did not evaluate the therapeutic alliance, which might also have influenced treatment responses [43]. Finally, we examined only the association between motivation to change and short-term outcome at discharge. Thus, it would be desirable to reexamine this sample after a longer time period.

There are also considerable strengths of our study, including the prospective study design and the consecutive recruitment of adolescent patients requiring hospital treatment. We used both self-reporting and interview-based instruments to assess eating disorder psychopathology, and we investigated motivation to change with respect to weight outcome at several time points during treatment.

## Conclusions

Motivation to change is an important predictor of short-term outcome with respect to weight gain trajectory in adolescent AN patients. A greater motivation to change at admission is followed by greater weekly weight gain. Interestingly, an initial reluctance to enter hospital treatment did not have a negative influence on the readiness to change, whereas patients with a less severe state of the disorder (in terms of BMI at admission) or a more severe eating disorder-specific psychopathology exhibited a lower motivation to change. To date, no available evidence-based intervention successfully addresses motivation to change in patients with eating disorders. Given the impact of this measure on the course of treatment, further research on how to enhance motivation to change in this patient group is urgently needed.
